# *pAo*: A Consensus Genome-Scale Metabolic Model for *Aspergillus oryzae* Capturing Intraspecies Diversity

**DOI:** 10.34133/csbj.0173

**Published:** 2026-08-19

**Authors:** Jeroen Gilis, Casper Robert Balten van der Luijt, Marilena Feller, Candela Sánchez-Girón Barba, Morten Otto Alexander Sommer, Leonie Johanna Jahn, Eduard Johannes Kerkhoven

**Affiliations:** ^1^ Department of Life Sciences, Chalmers University of Technology, 412 96 Gothenburg, Sweden.; ^2^ Department of Food Science, Faculty of Science, University of Copenhagen, 1958 Frederiksberg, Denmark.; ^3^ The Novo Nordisk Foundation Biotechnology Research Institute for the Green Transition, Technical University of Denmark, 2800 Kgs. Lyngby, Denmark.; ^4^ National Food Institute, Technical University of Denmark, 2800 Kgs. Lyngby, Denmark.; ^5^ Science for Life Laboratory (SciLifeLab), 412 96 Gothenburg, Sweden.

## Abstract

*Aspergillus oryzae* (koji mold) is a key microorganism in traditional food fermentations including soy sauce, sake, and miso and is important in novel culinary applications and modern biotechnology, such as sustainable meat alternatives and enzyme production. Despite its industrial importance, until recently, the most recent genome-scale metabolic model (GEM) for *A. oryzae* dated back to 2008 and was limited to a single strain (RIB40). Here, we present *pAo*, a pan-GEM for *A. oryzae*, integrating genomic data from 187 strains to capture species-wide metabolic diversity. Our model comprises 2,025 reactions, representing a 52% increase in metabolic coverage over the RIB40-based model and includes previously overlooked pathways, such as cytochrome P450-mediated xenobiotic metabolism and extended amino acid metabolism. Using this pan-GEM, we derived strain-specific GEMs and validated 8 of them through high-throughput phenotypic screening on 285 substrates. Growth experiments on 4 industrially relevant carbon sources revealed substantial interstrain metabolic diversity, although flux balance analysis indicated that this variability originates at the regulatory rather than network-structural level. This resource provides a foundation for informed strain selection for biotechnological applications and future metabolic engineering in *A. oryzae*.

## Introduction

*Aspergillus oryzae*, commonly known as koji mold, is a cornerstone of traditional food fermentations, such as soy sauce, sake, and miso. Because of its long history of safe use in the food industry, *A. oryzae* was granted the status of a Generally Recognized as Safe organism by the US Food and Drug Administration and the World Health Organization [1–3]. This has enabled its expansion into modern biotechnology as a leading cell factory. For decades, *A. oryzae* has been renowned for its high secretion of native enzymes such as amylases and proteases [4–8] and its excellent capacity for recombinant protein production [9–11]. It is also widely used for producing organic acids, such as malic acid, kojic acid, and gluconic acid [12–16]. More recently, microbial foods using *A. oryzae* as a source of edible mycelium for sustainable meat alternatives have emerged [17–22], and its potential as a prebiotic and a probiotic in human diets and animal feed was demonstrated [23–27].

This versatility has established *A. oryzae* as a pivotal microorganism in both food science and industrial biotechnology. However, fundamental research into the molecular components that govern its metabolism—genes, proteins, and metabolic pathways—is key to further optimizing its biotechnological use and novel culinary applications [28,29]. In this context, genome-scale metabolic models (GEMs) have proven particularly powerful [30,31]. A GEM is a knowledge base constructed from data on an organism’s genome, the links between genes, proteins, and reactions, as well as the stoichiometry of these reactions. By incorporating biological constraints, such as nutrient availability and protein localization within the cell, GEMs can be used to perform flux balance analysis (FBA), which predicts the flux through each metabolic reaction in the organism [32]. These predicted fluxes provide a computational model of cellular metabolism at the genome scale, enabling the prediction of phenotypes such as gene essentiality, growth rate, and metabolite production rates. In addition, GEMs can inform metabolic engineering strategies that modify the organism’s genome to enhance the production titer, rate, and yield of a metabolite of interest [33,34].

With continuous improvements in molecular biology and whole-genome sequencing, the information required for constructing GEMs has become increasingly abundant and more accurate. For model organisms such as *Escherichia coli* [35,36], *Saccharomyces cerevisiae* [37,38], *Caenorhabditis elegans* [39,40], and *Homo sapiens* [41–43], dedicated efforts have been made to continuously update and refine GEMs with the latest data. Beyond these canonical models, GEMs are increasingly being developed for nonmodel and industrially relevant organisms, including several filamentous fungi [44–48]. For *A. oryzae*, only 2 GEMs have been reported to date. The first, and most widely used, was published in 2008 [49]. More recently, an updated GEM was developed [50]. However, this model was specifically designed to provide a detailed representation of cordycepin metabolism. It was constructed using the GEM of the entomopathogenic fungus *Cordyceps militaris* as a template, supplemented with additional reactions from the 2008 *A. oryzae* model. Consequently, this updated GEM is not ideally suited to function as a comprehensive, general purpose knowledge base for *A. oryzae* metabolism.

Moreover, strain-specific [51–53] and cell-type-specific [54–57] GEMs have become available for model organisms, enabling the capture of intraspecies diversity in metabolism. However, GEMs developed for *A. oryzae* remain limited to only 2 strains: the laboratory strain RIB40 and the engineered strain AoCordy-T1 [49,50].

The first objective of this manuscript is to build a knowledge base for the metabolism of *A. oryzae* at the species level, which we refer to as the pan-GEM for *A. oryzae* (*pAo*). Secondly, we integrate genotype information from 187 strains to derive strain-specific GEMs based on *pAo*. We then perform a high-throughput phenotype characterization and growth experiment to validate these strain-specific models and to assess intraspecies metabolic diversity. Finally, we argue that this extensive set of strain-specific GEMs provides an ideal foundation for making informed decisions about which strain offers the most potential for cell factory development and food fermentation processes.

## Results

### Mapping the metabolic potential of *A. oryzae* with a pan-GEM

**Fig. 1.**
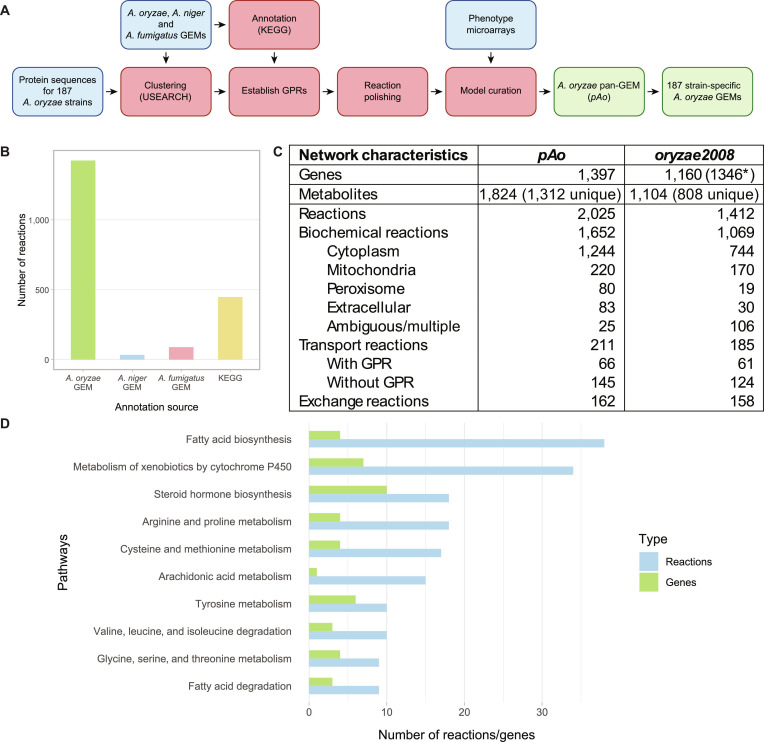
Reconstruction of a pan-genome-scale metabolic model (GEM) for *Aspergillus oryzae* (*pAo*). (A) Workflow for pan-GEM reconstruction. Protein sequences from 187 *A. oryzae* strains and 3 *Aspergillus* species with existing GEMs were clustered using USEARCH. Metabolic reactions were assigned on the basis of homology to known models and Kyoto Encyclopedia of Genes and Genomes (KEGG), followed by manual polishing of compartments, directionality, and metabolite names. The model was curated using phenotype microarray data, resulting in a pan-GEM. Strain-specific models were derived by subsetting the pan-GEM based on individual strain’s gene content. (B) Bar chart indicating the number of reactions coming from each of the different reaction databases used to construct the pan-GEM. (C) Network characteristics comparing the pan-GEM and *oryzae2008*, demonstrating the expanded coverage of genes, metabolites, and reactions in the pan-GEM. Genes are counted as the number of unique orthologous gene clusters as obtained from the USEARCH algorithm, while the value with the asterisks indicates the number of individual genes for *oryzae2008*. Metabolites present in multiple compartments are either counted separately or as one (unique). GPR, Gene–protein–reaction rule. (D) Bar chart displaying the number of reactions (blue) and genes (green) involved in the top 10 enriched pathways in *pAo* compared to the *oryzae2008* model.

We constructed a pan-GEM for *A. oryzae*, *pAo*, to capture the metabolic potential of the species. This model expands substantially upon the previous *A. oryzae* GEM (*oryzae*2008), which was limited to a single laboratory strain, RIB40 [49]. Our reconstruction pipeline integrated genomic data from 187 *A. oryzae* strains (Fig. 1A and Table S1). We predicted protein sequences and inferred their metabolic functions through homology searches against 3 curated *Aspergillus* GEMs (from *A. oryzae* RIB40, *Aspergillus niger*, and *A. fumigatus*) [46,49,58].

This analysis identified 11,949 orthologous protein sequence clusters, classified as strict core (present in all 187 strains), softcore (>95%), shell (5% to 95%), cloud (<5% but >1 strain), or strain specific (Fig. S1), comprising 6,959, 1,882, 1,687, 611, and 810 clusters, respectively. For clusters with orthologs in the established GEMs, we assigned the corresponding metabolic reactions. For the remaining clusters, we performed a BLAST [59] search against the Kyoto Encyclopedia of Genes and Genomes (KEGG) [60] reaction database to identify potential new metabolic functions.

The resulting model, *pAo*, comprises 2,025 reactions, with 1,426 obtained from *oryzae2008*, 86 incorporated from other *Aspergillus* GEMs, and 454 from KEGG (Fig. 1B). An additional 59 reactions were included as gap-filling based on experimental validation to ensure growth on specific substrates (Table S2). This curated network, catalyzed by 1,397 genes and encompassing 1,824 metabolites, serves as the *pAo* and substantially expands on the *oryzae2008* model (Fig. 1C) [49]. Most notably, the number of biochemical reactions (this excludes transport and exchange reactions) has increased from 1,069 in *oryzae2008* to 1,652 in *pAo*.

To assess the coverage of *pAo* relative to the previous *A. oryzae* GEM, we examined to what extent *oryzae2008* genes are represented in the *pAo* core. Of the 1,160 genes in *oryzae2008*, 69 are noncore (neither strict core nor soft core) in *pAo*; these genes catalyze 124 reactions in the template model, 30 of which relate to sterigmatocystin/aflatoxin biosynthesis, a pathway known to be strain variable and therefore not expected to form part of the *pAo* core [3,61,62]. Furthermore, although *pAo* is a superset of *oryzae2008*, *oryzae2008* lacks 49 genes that form part of the *pAo* core, which together catalyze 124 reactions, 26 of which relate to fatty acid biosynthesis (Fig. S2).

To facilitate downstream pathway analyses, each reaction was assigned a single pathway annotation according to the subsystem information from the KEGG database [60]. Comparing these annotations to *oryzae2008* revealed additional metabolic pathways in *pAo* that were likely overlooked in the earlier model (Fig. 1D). The most notable expansions were in fatty acid biosynthesis, comprising 38 reactions encoded by 4 genes, and in metabolism of xenobiotics (foreign compounds) by cytochromes P450—a superfamily of heme-containing monooxygenases [63]—comprising 34 novel reactions catalyzed by 7 genes. Although thoroughly documented in *A. oryzae* [64], this class of enzymes was absent in *oryzae2008*. We also observed 4 pathways involved in amino acid metabolism. While all enzymes that comprise the core of amino acid metabolism were already included in *oryzae2008*, our approach has identified 54 additional genes that extend amino acid metabolism in *A. oryzae*, for example, opening additional pathways for directing arginine metabolism toward butanoate or glyoxylate metabolism (Fig. S3).

### Strain-specific genome-scale models for *A. oryzae* reveal substantial intraspecies metabolic diversity

**Fig. 2.**
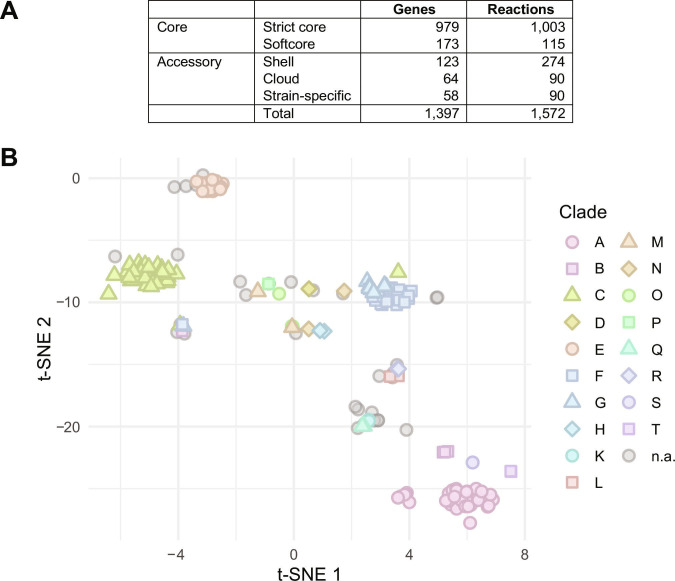
Analysis of 187 strain-specific *Aspergillus oryzae* genome-scale metabolic models (GEMs). (A) Conservation of gene–protein–reaction rule (GPR)-associated reactions and genes across the strain-specific models. Reactions and genes were classified by the percentage of strains in which they are present: strict core (100% of strains), softcore (>95% but not all strains), shell (5% to 95% of strains), cloud (<5% but more than one strain), and strain-specific (present in only one strain). (B) *t*-Distributed stochastic neighbor embedding (t-SNE) of the 187 strains based on the reaction pathway information. Strains are colored according to phylogenetic clade.

We used our pan-GEM to construct strain-specific GEMs for all 187 *A. oryzae* strains. Each model represents a subset of the reactions of the pan-GEM for which the corresponding genes are present in that specific strain. Spontaneous reactions and those with unknown genetic basis were included in all models. Analysis revealed a highly conserved metabolic core, with 1,152 genes (82.5%) and 1,118 gene–protein–reaction rule (GPR)-associated reactions (71.1%) present in more than 95% of strains (Fig. 2A). Despite this, we observed substantial strain-specific (90 GPR-associated reactions) metabolic content. Reduced-dimensional representation of the reactions present in each strain-specific GEM shows that *A. oryzae* strains cluster primarily according to their taxonomic clades (Fig. 2B), indicating that evolutionary relationships are reflected in metabolic network topology. This is corroborated by higher within-clade than between-clade reaction content similarity and by pairwise Jaccard similarity of reaction content (Figs. S4 and S5).

### High-throughput phenotyping drives model curation and reveals consistent substrate utilization patterns

**Fig. 3.**
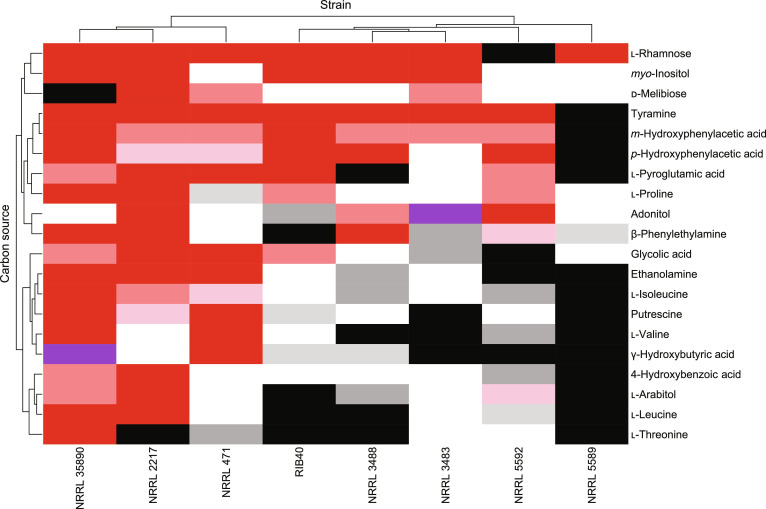
Heatmap for the metabolic activity measurements for 8 *Aspergillus oryzae* strains on the 20 carbon sources of the phenotype microarray experiment that display the largest intraspecies variability. Clustering was performed both on the strains and the substrates. Experiments were performed with 3 biological replicates, which have been collapsed into a single consensus call per strain–substrate combination. Coloring reflects both the consensus activity status and the agreement among replicates: Red indicates activity across all replicates; black indicates no activity across all replicates; light red indicates probable activity (2 active replicates); dark gray indicates probable no activity (one ambiguous or one active replicate among otherwise inactive replicates); pink or light gray indicates a possible but nonconclusive result; white indicates an ambiguous outcome; and purple flags contradictory replicate calls.

To assess phenotypic diversity, we performed high-throughput phenotyping of 8 phylogenetically diverse *A. oryzae* strains on 285 substrates (190 carbon and 95 nitrogen sources) on phenotypic microarrays (PMs). Clustering of the samples on the PMs indicated that biological replicates cluster together, confirming that interstrain differences exceed experimental variation (Fig. 3 and Fig. S6). Clustering all 285 substrates (Fig. S6) additionally revealed several coherent substrate clusters, particularly among nitrogen sources. For instance, one cluster contained key metabolites in arginine metabolism (putrescine, agmatine, and ornithine), and another group contained most of the tested dipeptides.

We then used the PM results to curate the 8 strain-specific GEMs. We selected 45 substrates that unambiguously supported metabolic activity across most replicates and performed FBA, using adenosine triphosphate (ATP) hydrolysis as a proxy for metabolic activity to specify the cellular objective function. For substrates on which FBA failed to predict metabolic activity, we systematically (a) assessed whether including a transport reaction for the substrates allowed for supporting metabolic activity, if appropriate; (b) assessed whether metabolic activity could be restored through gap-filling of reactions from the *A. oryzae* pan-GEM, and (c) manually inspected pathways involved in metabolizing the substrate to identify missing reactions and perform gap-filling. The latter was achieved by performing a manual literature search on the metabolism of the target compound in related fungi, further aided by a manual inspection of the metabolic pathway maps provided by KEGG. This led to the addition of 59 reactions to *pAo* (Table S2), ensuring that all 8 curated models accurately predict growth on the 45 validation substrates.

### Strain-specific substrate preferences guide optimal carbon source selection

**Fig. 4.**
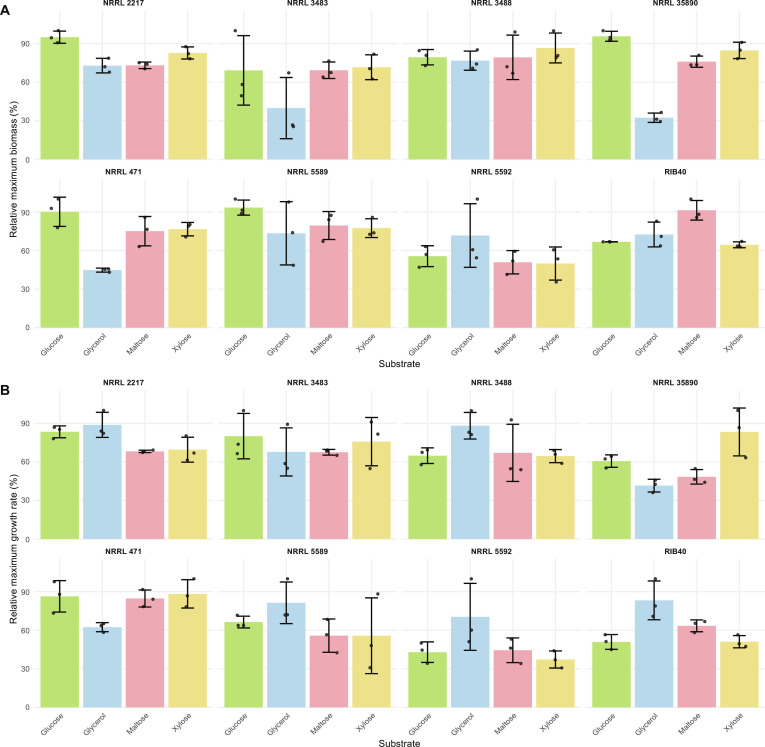
Comparison of growth between different substrates for 8 *Aspergillus oryzae* strains. (A) Biomass measurements. (B) Measurements of the maximum specific growth rate. Results are means ± SD of 3 biological replicates, normalized to the maximum value obtained for each strain.

To identify the optimal carbon source for each strain, we experimentally assessed the growth of the 8 phylogenetically diverse *A. oryzae* strains on 4 industrially relevant carbon sources: glucose, maltose, glycerol, and xylose (Table S3). Comparing biomass production and maximum growth rates for each strain across these substrates revealed distinct substrate preferences (Fig. 4 and Figs. S7 and S8). For instance, strain NRRL 35890 showed exceptionally high growth rates on xylose but slower growth on glycerol, a pattern also observed for NRRL 471. In contrast, strains NRRL 3488, NRRL 5592, and RIB40 appeared to favor glycerol as a substrate, achieving higher growth rates. These results provide a practical guide for selecting the best carbon source to maximize growth for a given strain, which is crucial for industrial applications where strain identity is fixed.

### Substrate-specific strain performance informs selection of optimal strains for industrial substrates

**Fig. 5.**
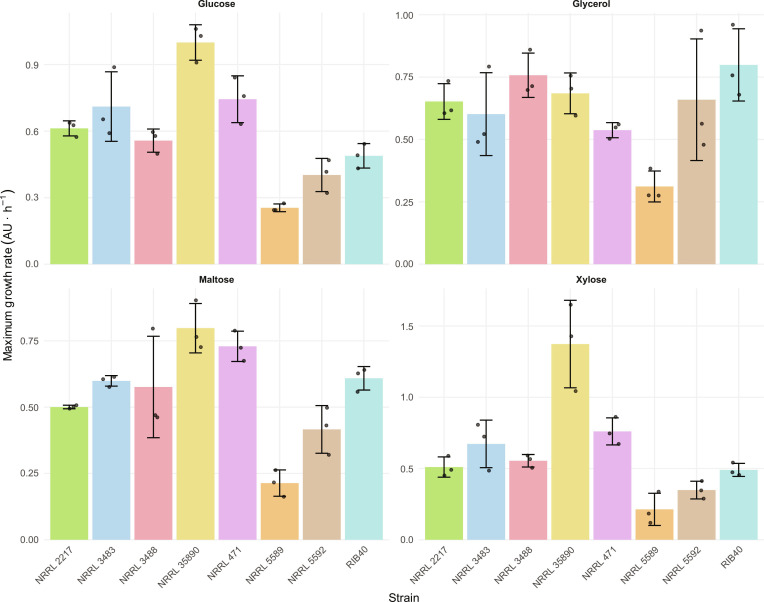
Bar chart for comparing the growth rates of 8 *Aspergillus oryzae* strains on different carbon sources. Measurements were obtained for 3 biological replicates on glucose, maltose, glycerol, and xylose. Results are means ± SD of 3 biological replicates, expressed in arbitrary units (AU).

Next, we evaluated which strain performs best on each carbon source to inform strain selection for industrial applications. We observed substantial interstrain variation in maximum growth rates across all carbon sources (Fig. 5). Strain NRRL 5589 showed consistently slow growth rates, while NRRL 35890 was consistently among the fastest. However, performance was highly substrate dependent. For example, on xylose, NRRL 35890 outperformed other strains, whereas on glycerol, NRRL 2217, NRRL 3488, NRRL 5592, and RIB40 were top performers alongside NRRL 35890. This highlights that strain selection can be tailored to the intended substrate to optimize utilization and growth rates.

To place the observations from these growth experiments in context, we compared our data with that used by Vongsangnak *et al.* [49]. It should be noted that although their model was developed for strain RIB40, the underlying experimental data originated from a study on another strain (A1560), and the reported xylose yield was inferred from a study on *A. niger* and *Aspergillus nidulans* [49,65,66]. Accordingly, for comparative purposes, we used overall mean values of maximum growth rate and biomass across all strains to represent the species as a whole.

In their study, biomass yields show minimal variation after carbon normalization, whereas our data indicated comparable maximum biomasses across all substrates except glycerol, which supported lower biomass production (Fig. S9). Furthermore, while Vongsangnak *et al*. [49] reported that *A. oryzae* achieved the highest maximum growth rate on glucose, followed by maltose, glycerol, and xylose, our experiments did not reveal a clear hierarchy among carbon sources (Fig. S10). Altogether, these results emphasize the need for substrate-specific strain evaluation.

### Flux analysis reveals a conserved metabolic solution space across the strain-specific models

**Fig. 6.**
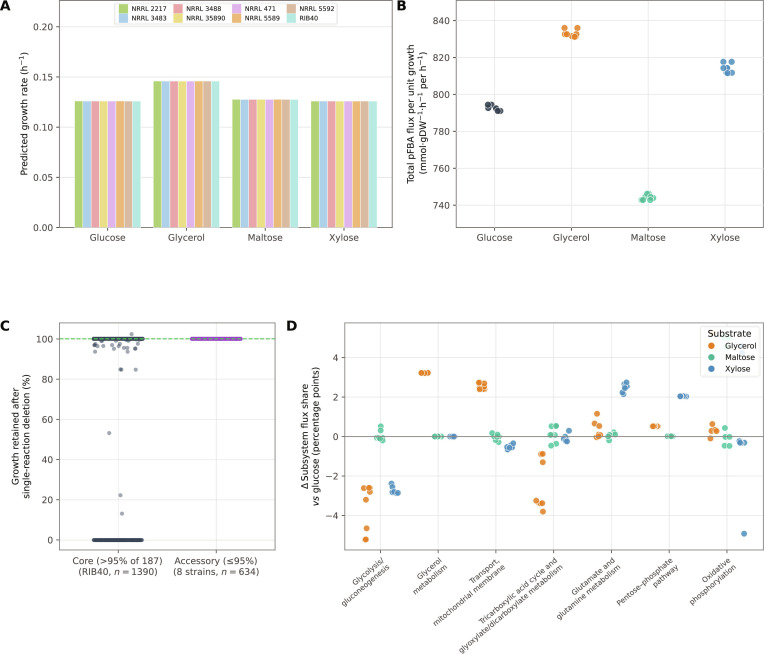
Comparative flux analysis of 8 strain-specific *Aspergillus oryzae* genome-scale metabolic models (GEMs) across 4 carbon sources. (A) Flux balance analysis (FBA)-predicted maximum growth rate per strain, with carbon uptake normalized to an equal carbon mole supply. (B) Parsimonious FBA (pFBA) costs, as total flux per unit growth, per strain. (C) Growth retained after single-reaction deletion of core and accessory reactions. The core simulations were done in the RIB40 GEM, and the accessory reactions were done in the relevant strain-specific GEM. (D) Change in subsystem flux share relative to glucose for the 7 most-affected subsystems; each point is one strain, colored by substrate.

The growth experiments revealed substantial interstrain variation in growth across the 4 carbon sources (Fig. 5). To assess whether the strain-specific models reproduced this phenotypic diversity, we used them to simulate growth by FBA. The models predicted identical biomass yields for all 8 strains and thus did not capture the experimentally observed variation (Fig. 6A). To establish whether this reflected a genuine equivalence of the reconstructed networks, rather than merely the limited discriminatory power of a single optimal solution, we characterized the metabolic solution space of the 8 curated models in detail.

Parsimonious FBA (pFBA) showed that, under each carbon source, the 8 models converged on an essentially identical optimal flux state. The predicted biomass yield per carbon mole, the ATP turnover per unit biomass, and the total flux required per unit growth (Fig. 6B) each varied by less than 0.3% across strains, whereas the differences between substrates were an order of magnitude larger and consistent across every strain (Fig. 6B). The carbon source, rather than strain identity, therefore determined the simulated metabolic operating point.

Systematic single-reaction deletion identified 169 reactions whose removal abolished growth, all belonging to the conserved core; in contrast, none of the 634 accessory reactions, deleted individually in each strain that encodes it, reduced the predicted growth rate by more than 0.06% (Fig. 6C). Loopless flux variability analysis (FVA) showed that, of the 827 reactions able to carry flux, 650 (79%) had an attainable flux range that was identical across strains and the comparatively few reactions that did differ were predominantly core reactions whose feasible direction or magnitude shifted. These variable range reactions concentrated in the functionally redundant fatty acid synthase cycle, peripheral sugar catabolism, and a small number of intercompartmental transporters, rather than accessory genes (Fig. S11). Because these reactions are not used at the parsimonious optimum, the interstrain variability resided in unused network capacity and did not affect the growth-supporting flux.

Finally, the substrate-specific rewiring of metabolism was itself consistent across strains. The change in flux distributed to each metabolic subsystem when switching from glucose to glycerol, maltose, or xylose was highly reproducible between the 8 strains (maximum interstrain standard deviation ~3 percentage points; Fig. 6D), confirming that the conserved core, and not strain-specific gene content, governed the predicted response to each carbon source. Together, these analyses show that the strain-specific models do not differ appreciably in the solution space probed by the tested growth conditions, which both explains the limited interstrain resolution of FBA and localizes the experimentally observed phenotypic diversity to regulatory rather than network-structural differences.

## Discussion

In this study, we deliver 3 key resources to advance the metabolic modeling of the industrially important mold *A. oryzae*: first, a comprehensive pan-GEM (*pAo*), representing the full metabolic potential of 187 *A. oryzae* strains; second, a corresponding collection of 187 strain-specific GEMs derived from this pan-GEM; and, third, a set of 8 highly curated models for phylogenetically diverse strains, validated against high-throughput PM data. The *pAo* model serves as a state-of-the-art catalog that captures a considerably higher number of reactions and metabolites than the previous *oryzae2008* model. Its primary use lies in its function as a template, which streamlines the generation of strain-specific models by enabling automated gap-filling and allowing for the rapid annotation of new strains via straightforward homology searches. Furthermore, the pan-GEM can be used to assess the overall metabolic capacity of *A. oryzae* for a specific biotechnological application and subsequently for the identification of the most suitable strain. We envision this resource platform will facilitate the consistent metabolic annotation of novel *A. oryzae* strains and enable iterative updates as new genomic data becomes available.

To validate our approach, we selected 8 strain-specific GEMs from diverse phylogenetic clades for experimental curation. These strains were chosen to span the major phylogenetic diversity within *A. oryzae*, which we show is reflected in substantial differences in metabolic network topology, enabling representative validation across evolutionary lineages while maintaining experimental feasibility given the inherently lower throughput of filamentous fungal systems compared to bacterial or yeast models. High-throughput phenotyping on 285 substrates identified 45 that unambiguously supported metabolic activity across most of the 8 selected strains. We curated the models to ensure that they predicted growth on these substrates. A conscious decision was made not to curate models based on a lack of metabolic activity, as this phenotype cannot be unambiguously translated into reaction-level constraints. While removal of specific individual reactions may mimic the observed nongrowth phenotype *in silico*, it would not be possible to reliably identify true absent reactions due to baseline signal that impedes nongrowth identification, regulatory effects that may be alternative sources of nongrowth, and metabolic redundancy. We also encountered a challenge with substrates showing ambiguous or strain-dependent activity. For these, defining a universal cutoff to separate true metabolic capability from noise was problematic; future work could use a sliding window analysis across multiple activity thresholds to resolve these cases, which would further enhance model heterogeneity and predictive accuracy.

A key finding from this validation highlighted a fundamental challenge in metabolic modeling: While our experiments revealed strain-specific differences in biomass production on 4 carbon sources, pFBA and FVA failed to predict this variation. The model simulations rather showed that 8 strain-specific models occupy essentially the same flux solution space under the tested conditions, with strain-to-strain differences in predicted yield and energetic cost below 0.3% and with no accessory reaction individually affecting the growth optimum. The reactions that distinguish the strains lie predominantly in peripheral, functionally redundant capacity that is not carried at the optimum. The conserved metabolic core dictates the simulated phenotype, and the substantial growth differences measured experimentally must originate at the regulatory or kinetic level rather than in metabolic network structure. This reframes the limited interstrain resolution of FBA not as a shortcoming of the reconstructions but as a structural property of the species’ metabolism, and it reinforces the case for enzyme- or proteome-constrained extensions [67] to capture the regulatory layer. This limitation is further compounded by the absence of systematically curated gene essentiality datasets for *A. oryzae*. Existing knockout studies are fragmented and inconsistently annotated, limiting their direct integration into large-scale model validation workflows. While genome-wide deletion collections are available for *S. cerevisiae* [68] and *Neurospora crassa* [69,70] and have been used for curation of GEMs [52,71,72], comparable resources are not yet available for *A. oryzae*. Their development would provide a valuable foundation for future constraint-based model validation and refinement.

When comparing our work to previous studies, methodological differences are crucial to consider. For instance, discrepancies between our growth data for the RIB40 strain and the study by Vongsangnak *et al.* (which presented the o*ryzae2008* model) [49] likely stem from differing experimental setups. Our biomass production and growth rates were determined in 24-well plates, based on arbitrary units, whereas their data are compiled from various experiments on strain A1560 rather than RIB40: growth rates from batch fermentations in 1.2 l of bioreactors and biomass yields from 1.5 l of chemostats [65]. Furthermore, their reported biomass yield on xylose was inferred from a study on *A. nidulans* and *A. niger* [66], not *A. oryzae.*

Furthermore, a single biomass equation was used for all models generated in this study, adopted directly from *oryzae2008* [49], which, in turn, was based on experimental data from various studies [73–76]. The use of a single biomass composition across strains is common practice in fungal genome-scale metabolic modeling [45,49,77,78], although biomass compositions are known to (partially) vary with growth conditions and genetic background [74,78]. While strain-specific biomass composition measured under a defined growth condition could refine model accuracy, characterizing every strain represented by a pan-GEM such as *pAo* would require substantial additional experimental work, even if many of the resulting compositions are anticipated to be comparable across phylogenetically related strains. This limitation may particularly be relevant for biomass yield predictions [78], where FBA was not able to fully capture the experimentally observed strain-specific variations in this study. However, this discrepancy likely arises not only from the use of a fixed biomass composition but also from the more general limitations of using conventional GEMs, which do not account for regulatory effects or kinetic constraints.

Concurrently with our study, another GEM, *iNR1684*, was developed for *A. oryzae* AoCordy-T1, a derivative of the strain BCC7051 engineered for cordycepin production [50]. This model was based on homology to a model for *C. militaris* (*iNR1329*), incorporated *oryzae2008*, and was gap-filled using GEMs from various other fungi. This approach resulted in a model highly specialized for a specific strain and biosynthetic pathway. In contrast, our pan-GEM provides a comprehensive, species-wide foundation that enables the consistent generation and comparison of models across *A. oryzae* strains, making it a broadly applicable resource for the community. Notably, compared to the *iNR1684* model, *pAo* contains 274 additional biochemical reactions and 153 more metabolites, highlighting the advantage of a pan-genome-based model in capturing the metabolic capabilities of the species (Table S4).

In conclusion, we present a collection of curated GEMs for *A. oryzae* based on a pan-GEM, substantially expanding on previous models, but with limited representation of phenotypic intraspecies variability. A promising strategy to overcome this limitation is incorporating additional layers of information into the FBA simulation workflow. We anticipate that in the future, our pan-GEM will be extended to include more *A. oryzae* strains and enhanced with such data, following the same trend observed for model organisms such as *S. cerevisiae*. Specific implementations could include imposing enzyme constraints [79], developing a proteome-constrained genome-scale protein secretory model [80], or expanding the compartmental resolution of *pAo* beyond the current 4-compartment structure. Such enhancements would directly address the current limitations in predicting phenotypic expression and would substantially expand the utility of our pan-GEM for the research community, given *A. oryzae*’s widespread use as a host for recombinant protein production.

## Methods

### Strain selection

The comprehensive genomic dataset used in this study is described in detail in van der Luijt *et al.* (in preparation). Briefly, we combined genome assemblies from newly sequenced *A. oryzae* NRRL strains and A1560 with publicly available genomes from National Center for Biotechnology Information (NCBI)’s Genome database [81] and the comprehensive *A. oryzae* genome database across the clades [82]. In addition, we included *de novo* assemblies from raw sequencing data obtained from NCBI’s Sequence Read Archive [83]. All genomes were assessed for completeness using BUSCO (v5.8.2) [84,85], and gene prediction was performed with AUGUSTUS (v3.4.0) [86]. To ensure high-quality data, we retained only genomes with a BUSCO completeness score of >94%. Clade assignments are also described in detail in van der Luijt *et al.* (in preparation).

### Constructing the *A. oryzae* pan-GEM

GPRs were established to link each protein sequence to the metabolic reactions it catalyzes. This is accomplished by associating the novel protein sequences with those of known GPRs through orthology. Specifically, we use protein sequences from 3 well-curated GEMs: the *oryzae2008* GEM of *A. oryzae* RIB40 [49] and 2 GEMs from the closely related species *A. niger* [58] and *Aspergillus fumigatus* [46].

To group homologous sequences into clusters of orthologous genes, ensuring that duplicated or highly similar genes are represented as a single gene rather than counted multiple times, protein sequence clustering is performed using BPGA [87], which uses the *clust_fast* function from the USEARCH algorithm [88]. Sequences were grouped into clusters of orthologous genes using an identity threshold of 0.5. Importantly, this clustering step does not itself determine gene function; GPRs are subsequently assigned to novel sequences through guilt by association with the established GEMs.

This threshold of 0.5 was chosen because it is (a) permissive enough to link a larger fraction of novel sequences to those in the 3 established GEMs, while (b) stringent enough to ensure that most sequences from the established GEMs cluster separately. For instance, of the 1,346 proteins annotated with a GPR in *oryzae2008*, 1,160 sequence clusters were identified using these settings. In total, this approach resulted in the identification of 11,949 protein sequence clusters.

For our pan-GEM, we adopt the same 4 cellular compartments described in the *oryzae2008* GEM: cytoplasm, mitochondria, peroxisome, and extracellular space. In contrast, the *A. niger* and *A. fumigatus* GEMs also assign reactions to additional compartments, including the nucleus, Golgi apparatus, endoplasmic reticulum, vacuole, and lipid particles. To ensure consistency across template models, reactions from these compartments were all assigned to the cytoplasm in our pan-GEM. Compartmentalization matters in GEMs, but the reactions most influential for phenotypic prediction belong to energy metabolism and (branched chain) amino acid metabolism [89], both localized to the retained mitochondrion. The collapsed organelles are dominated by lipids and are already connected by transport reactions representing vesicle shuttling, so collapsing these few compartments should not detrimentally affect predictions. In addition, we note that the 3 established GEMs use different nomenclatures for metabolites. To address this, we consolidated the original metabolite names using the KEGG [60], ChEBI [90], and PubChem [91] databases. Metabolite identifiers were standardized to KEGG identifiers when available, ChEBI identifiers if no KEGG identifier could be obtained, and metabolite names when neither identifier was found.

For sequences without orthologs in any of the 3 established GEMs, we perform a BLAST [59] homology search to the KEGG [60] reaction database using the *getKEGGModelForOrganism* function from the RAVEN toolbox [92]. This function, designed to reconstruct a GEM based on protein homology to orthologs of all eukaryotic organisms cataloged in KEGG (KEGG release 105), allows us to retrieve the GPRs for sequences lacking orthologs. Subsequently, we remove any additional reactions that were included by *getKEGGModelForOrganism* to report a standalone GEM. The cellular localization of the remaining reactions is then predicted using DeepLoc [93]. Finally, since the KEGG reaction database typically lacks accurate information on reaction reversibility, we update the reversibility of reactions using the online equilibrator tool [94] (https://equilibrator.weizmann.ac.il). Sequences for which no GPRs could be established using any of these methods were excluded from the pan-GEM.

The biomass equation was adopted directly from *oryzae2008* (Table S5) [49]. The absence of erroneous energy-generating cycles was evaluated by testing whether dissipation reactions of the following energy carriers were zero: ATP, cytidine triphosphate, guanosine triphosphate, uridine triphosphate, inosine triphosphate, reduced nicotinamide adenine dinucleotide (NADH), reduced nicotinamide adenine dinucleotide phosphate (NADPH), reduced flavin adenine dinucleotide (FADH_2_), reduced flavin mononucleotide (FMNH_2_), acetyl-coenzyme A, l-glutamate, and ubiquinol-8, in accordance to the approach described by Fritzemeier *et al* [95]*.* Three such cycles initially present in the pan-GEM, arising from reactions adopted from other *Aspergillus* species, were eliminated by constraining 4 reactions (OtherAsp_R04962, OtherAsp_R01708, r1736, and r1737) to their thermodynamically feasible direction.

The model quality was assessed by *memote* [96]. The consensus *pAo* model scored 57.7% overall, with all reactions charge-balanced and ~80% mass-balanced (limited by ~430 reactions with generic/polymeric pseudometabolites) that cannot carry a unique formula. Full reports are available at https://doi.org/10.5281/zenodo.18598684.

### Model similarity analysis for strain-specific GEMs

We used the *compareMultipleModels* function from RAVEN toolbox [92] to compare the strain-specific GEMs at the reaction level. Briefly, a binary reaction existence matrix was collected based on which pairwise Hamming distances between strains are calculated. These distances were used as input for *t*-distributed stochastic neighbor embedding (t-SNE) to visualize the strain-specific GEMs in reduced dimension space.

### Strains and media

Eight *A. oryzae* strains (RIB40, NRRL 471, NRRL 2217, NRRL 3483, NRRL 3488, NRRL 5589, NRRL 5592 and NRRL 35890) were used for phenotypic characterization in 96- and 24-well plates.

Potato dextrose agar (glucose [20 g/l], potato infusion [4 g/l], and agar [15 g/l]) plates and slants were used to initially revive strains from conidial suspension stored as cryostocks in 20% glycerol at −70 °C and harvest conidia.

For PM assays, filamentous fungi inoculating fluid (Biolog Inc., Hayward, CA, USA) was used as base medium, supplemented differently depending on the PM plate type: no supplements for PM1 and PM2A plates and 100 mM d-glucose, 5 mM potassium phosphate monobasic anhydrous, and 2 mM sodium sulfate for PM3B plates.

For 24-well plate growth studies, a Czapek–Dox medium (NaNO_3_ [3 g/l], KCl [2 g/l], KH_2_PO_4_ [1 g/l], MgSO_4_·7H_2_O [0.5 g/l], and FeSO_4_ [0.02 g/l] [pH 5.5]) was prepared. This medium was supplemented with 20 g/l of one of 4 carbon sources (glucose, maltose, glycerol, or xylose), 0.01% (v/v) Tween 80 as a surfactant, and chloramphenicol [25 μg/ml] to prevent bacterial contamination.

### Phenotypic microarrays

PM experiments were conducted using PM1, PM2A, and PM3B plates (Biolog Inc.). Each well was inoculated with 100 μl of a fresh conidial suspension prepared in filamentous fungi inoculating fluid with the appropriate supplements, dispensed using an Opentrons OT-2 automated pipette (Opentrons Labworks Inc., Long Island City, NY, USA). Redox Dye D (Biolog Inc.), a tetrazolium-based dye that indicates metabolic activity via NAD(P)H-dependent reduction, was added to each well. Plates were incubated at 30 °C in an OmniLog multiple plate reader (Biolog Inc.) for 96 h, with metabolic activity monitored kinetically. All experiments were performed in biological triplicates.

### Analysis of phenotypic microarray data

Raw data from the PMs, stored as D5E files, was imported in Data Analysis 1.7.1.58 (Biolog Inc.) and subsequently exported as CSV files. The CSV files were imported into R 3.5.1 and processed with the *opm* package (v1.3.77), using the custom R script *opm_pipeline*.

To construct the heatmaps of Fig. 3 and Fig. S6, we used the raw data from the PM experiment, i.e., metabolic activity measurements along 96 h taken at 1-h time intervals for 8 strains in biological triplicate. As a measure for metabolic activity for a specific replicate of a strain, we take the metabolic activity at the last time point and subtract the average of the 10 media with the lowest metabolic activity on the same plate at the last time point. While a negative control sample is included on each plate for the purpose of normalization, we found that, in a few cases, these were noisy, and, therefore, we instead rely on the assumption that each plate contains at least 10 media for which truly no metabolic activity is observed. Finally, the heatmaps were constructed in R using the *complexHeatmap* package (v2.24.1) [97].

### Growth characterization

For growth characterization, 2.5 ml of modified Czapek–Dox medium (prepared as described above) was dispensed into each well of a 24-well plate (CR1424dg, Enzyscreen B.V., Heemstede, The Netherlands). Fresh conidial suspensions were used to inoculate the wells, and plates were incubated at 30 °C with shaking at 200 rpm in a Growth Profiler 960 (Enzyscreen B.V.) for 72 h. Growth was monitored over time, and each condition was tested in biological triplicates.

### Analysis of 24-well plate cultivation data

Images captured by the Growth Profiler 960 were analyzed using GP960Viewer software (Enzyscreen B.V.). The 24Wells_Square profile was applied with default multiwell plate dimensions, alongside the following custom parameters: spot size in *X* and *Y* directions (14 mm each), geometric correction (0.5 mm), and exclusion of the top 5% and bottom 75% of pixel intensity values to minimize medium and edge artifacts.

For each well, green intensity values were exported to a CSV file. Time points were converted to hours, and raw intensity values were blank corrected by subtracting the average of the 5 minimum values observed in the respective well. The blanked data were then analyzed using a custom R pipeline to fit splines using function *smooth.spline*, with smoothing set to spar = 0.7, to estimate (pseudo)growth rates from time-dependent changes in green value, taking the steepest section as maximum growth rate (Figs. S12 to S15).

### Simulating metabolism using FBA

To validate the 8 strain-specific GEMs, we used metabolic activity measurements on various carbon and nitrogen sources from the PM experiment. Each model was curated using strain-specific FBA [98], applied to metabolites where the PM data indicated strong metabolic activity. To run FBA on multiple models simultaneously in an efficient manner, we used the Python package *Medusa* [99]. Media were selected for curation based on the following criteria. First, we estimated background metabolic activity for each strain replicate by averaging the ten metabolites with the lowest activity on the corresponding plate at the final time point. A metabolite was included for downstream analysis if it met one of 2 conditions: (a) At least 3 strains had at least 2 replicates with an activity of ≥150 units above background, or (b) at least 2 strains had at least 2 replicates with an activity of ≥150 units above background, and at least 4 additional strains had at least 2 replicates with an activity of ≥100 units above background. With these criteria, 45 metabolites were retained for running FBA, and 240 were excluded. If FBA predicted no growth for a strain-specific GEM for a metabolite that clearly supported metabolic activity in the PM experiment, we curate the GEM by (a) automatically gap-filling reactions from the pan-GEM, (b) adding a transport reaction for the target metabolite if appropriate, or (c) by manual gap-filling. After this round of model validation, strain-specific FBA growth predictions correspond perfectly with the observed metabolic activity measurements of the PM experiment for all 45 selected metabolites.

To compare with experimental growth data, FBA and FVA were performed with each carbon source supplied at an equal carbon flux of 6.72 mmol C·gDW^−1^·h^−1^, corresponding to the measured specific glucose uptake rate (*q*_S_ = 1.12 mmol glucose·gDW^−1^·h^−1^; Table S5), with a nongrowth associated ATP maintenance of 1 mmol·gDW^−1^·h^−1^. FVA was run at 90% of the maximal growth rate under a loopless constraint. Reactions differing most between strains were identified by ranking them by the variance, across the 8 strains of their loopless flux range on glucose.

## Data Availability

The code for reproducing all analyses and figures and the final GEM for *A. oryzae* are shared on GitHub (https://github.com/SysBioChalmers/panAsp-GEM). All code, models, and data used and generated in this project can be retrieved from Zenodo (10.5281/zenodo.18598684).

## Supplementary Materials

Supplementary 1 Figs. S1 to S15 Tables S1 to S5 References [100–120]
