# Supplementary material for: pAo: A Consensus Genome-Scale Metabolic Model for Aspergillus oryzae Capturing Intraspecies Diversity
Source: Comput Struct Biotechnol J. 2026 Aug 19;35(1):0173. doi: 10.34133/csbj.0173 (PMC13486731; doi:10.34133/csbj.0173)
Supplement: Supplementary file 1 [file csbj.0173.f1.docx]

# *pAo*: a consensus genome-scale metabolic model for *Aspergillus oryzae* capturing intra-species diversity

Jeroen Gilis^1*^, Casper R.B. van der Luijt^2,3,4*^, Marilena Feller^3^, Candela Sánchez-Girón Barba^3^, Morten O.A. Sommer^3^, Leonie J. Jahn^3†^, Eduard J. Kerkhoven^1,3,5†^

1: Department of Life Sciences, Chalmers University of Technology, Kemigården 1, 412 96 Gothenburg, Sweden

2: Department of Food Science, Faculty of Science, University of Copenhagen, Rolighedsvej 26, 1958 Frederiksberg, Denmark

3: The Novo Nordisk Foundation Biotechnology Research Institute for the Green Transition, Technical University of Denmark, Søltofts Plads 220, 2800 Kgs. Lyngby, Denmark

4: National Food Institute, Technical University of Denmark, Henrik Dams Allé 202, 2800 Kgs. Lyngby, Denmark

5: Science for Life Laboratory (SciLifeLab), 412 96 Gothenburg, Sweden

*: These authors contributed equally

†: Corresponding author

#### Keywords

*Aspergillus oryzae*, genome-scale metabolic model (GEM), pan-genome, metabolic diversity, strain-specific modelling

## Supplementary material

**Supplementary Figure S1.** **Gene cluster partitions of the *Aspergillus oryzae* pan-genome across 187 strains, used to construct the pan-genome-scale metabolic model *pAo*.** The donut chart shows the number of orthologous gene clusters assigned to each pan-genome partition: strict core (present in 100% of strains), softcore (present in >95% but not 100% of strains), shell (present in 5–95% of strains), cloud (present in <5% but more than one strain), and unique/strain-specific (present in only one strain). The total number of orthologous gene clusters in *pAo* is 11,949.

| **A** |  |
| --- | --- |
| **B** |  |

**Supplementary Figure S2. Distribution of non-core gene content across 187 strain-specific genome-scale metabolic models of *Aspergillus oryzae*. A. Distribution of non-core (clusters of orthologous) genes across *A. oryzae* models.** Most models contain between 35 and 65 non-core genes. Non-core (accessory) genes are defined as genes that occur in fewer than 95% of the strain-specific models. **B. Distribution of reactions catalysed by non-core genes across *A. oryzae* models.** The distribution of reactions is broader than that of genes (panel A), reflecting variability in the metabolic capabilities encoded by non-core genes.

**Supplementary Figure S3.** **Snapshot of the “Arginine and proline metabolism” metabolic map (map00330) from the KEGG database.** Reactions that were newly added in our pan-GEM compared to the oryzae2008 model are indicated in red. Note that some of these reactions facilitate directing arginine metabolism towards butanoate or glyoxylate metabolism.

**Supplementary Figure S4. Within- vs. between-clade reaction-content similarity.** Boxplots of pairwise Jaccard similarity of reaction content between strain-specific *Aspergillus oryzae* GEMs, comparing strain pairs belonging to the same clade (within) or different clades (between). Within-clade similarity is consistently higher than between-clade similarity across all clades, indicating that metabolic network composition is more conserved among evolutionarily related strains. Strain not assigned to any clade (NA), which are distributed across the *A. oryzae* phylogeny, show Jaccard similarity values comparable to between-clade pairs.

**Supplementary Figure S5. Pairwise Jaccard similarity of reaction content across 187 strain-specific *Aspergillus oryzae* GEMs**. Strains are ordered by hierarchical clustering, with clade membership indicated by color-coded labels. High-similarity blocks along the diagonal correspond to groups of strains within the same clade, reflecting the conservation of metabolic network topology across evolutionarily related strains.

| **A** |  | **B** |  |
| --- | --- | --- | --- |

**Supplementary Figure S6. Heatmap for the metabolic activity measurements for eight *Aspergillus oryzae* strains on all 285 substrates of the BioLog phenotype microarray experiment. A. Carbon sources (PM01 and PM02A plates). B. Nitrogen sources (PM03B plates).** Clustering was performed both on the strains and the substrates. Experiments were performed with three biological replicates, which have been collapsed into a single consensus call per strain–substrate combination. Colouring reflects both the consensus activity status and the agreement among replicates: red indicates activity across all replicates; black indicates no activity across all replicates; light red indicates probable activity (two active replicates); dark gray indicates probable no activity (one ambiguous or one active replicate among otherwise inactive replicates); pink or light gray indicates a possible but non-conclusive result; white indicates an ambiguous outcome; and purple flags contradictory replicate calls.

**Supplementary Figure S7. Comparison of growth between different substrates for eight *Aspergillus oryzae* strains, biomass measurements.** Results are mean ± standard deviation of 3 biological replicates, expressed in arbitrary units.

**Supplementary Figure S8. Comparison of growth between different substrates for eight *Aspergillus oryzae* strains, growth rates.** Results are mean ± standard deviation of 3 biological replicates, expressed in arbitrary units.

**Supplementary Figure S9. Species-level maximum biomass obtained by *Aspergillus oryzae* across different substrates.** Results are mean ± standard deviation of 24 cultivations, expressed in arbitrary units.

**Supplementary Figure S10. Species-level maximum growth rates achieved by *Aspergillus oryzae* across different substrates.** Results are mean ± standard deviation of 24 cultivations, expressed in arbitrary units.

**Supplementary Figure S11. Flux ranges from loopless FVA in eight different *Aspergillus oryzae* strains.** After constraining each model with the same glucose uptake rate, the reaction-specific flux ranges are shown, grouped by subsystem. While isolated differences exist across models, most differences are in fatty acid biosynthesis and mitochondrial membrane transport.

**Supplementary Figure S12. Spline fits of raw Growth Profiler growth data for 24-well plate cultivations of eight *Aspergillus oryzae* strains on glucose.** Results are individual spline fits (coloured) and mean spline fit (black) of 3 biological replicates.

**Supplementary Figure S13. Spline fits of raw Growth Profiler growth data for 24-well plate cultivations of eight *Aspergillus oryzae* strains on glycerol.** Results are individual spline fits (coloured) and mean spline fit (black) of 3 biological replicates.

**Supplementary Figure S14. Spline fits of raw Growth Profiler growth data for 24-well plate cultivations of eight *Aspergillus oryzae* strains on maltose.** Results are individual spline fits (coloured) and mean spline fit (black) of 3 biological replicates.

**Supplementary Figure S15. Spline fits of raw Growth Profiler growth data for 24-well plate cultivations of eight *Aspergillus oryzae* strains on xylose.** Results are individual spline fits (coloured) and mean spline fit (black) of 3 biological replicates.

**Supplementary Table S1. Strains and genomes used in this study.** List of genomes used in this study and metadata on strain isolation. All strains are *Aspergillus oryzae*. Clade assignments originate from van der Luijt, *et al*. (in preparation), ‘-’ denotes unknown clade assignment.

| Strain ID | Country / region of isolation | Isolation source / use | Clade | Genome data source | Reference(s) |
| --- | --- | --- | --- | --- | --- |
| 3.042 | China | soil | K | GCA_000269785.2 | ^100^ |
| 14160 | China | koji | A | GCA_019097755.1 | ^101^ |
| 100-8 | China | soil/mutant | K | GCA_000691885.1 | ^102^ |
| A1560 | Japan | koji | F | TBD | ^103^ |
| AS 3.863 | China | soy sauce | - | GCA_000278425.1 | ^104^ |
| AS 3.951 | China | soy sauce/mutant | - | GCA_000278405.1 | ^104^ |
| BCC7051 | Thailand | soil | E | GCA_002007945.1 | ^105^ |
| BP2-1 | South Korea | nuruk | - | GCA_004353305.1 | ^106^ |
| CAM-B | Japan | rice koji | A | GCA_040954775.1 | ^107^ |
| CBS126860 | India | soil | - | CAoGDX | ^82,108^ |
| ISS-B | Japan | rice koji | A | GCA_040954735.1 | ^107^ |
| ISS-M | Japan | rice koji | A | GCA_040938365.1 | ^107^ |
| ISS-T | Japan | rice koji | A | GCA_040938235.1 | ^107^ |
| KBH-B | Japan | rice koji | A | GCA_040954795.1 | ^107^ |
| KBP3 | South Korea | nuruk | - | GCA_008032055.1 | ^109^ |
| KDG21 | South Korea | nuruk | - | GCA_018140735.1 | ^109^ |
| KDM1 | South Korea | doenjang meju | L | GCA_039654725.1 | ^81^ |
| KG15 | South Korea | nuruk | - | GCA_013407045.1 | ^109^ |
| KJJ4b | South Korea | nuruk | - | GCA_018140755.1 | ^109^ |
| KSS2 | South Korea | nuruk | - | GCA_008032255.1 | ^109^ |
| KSW16 | South Korea | nuruk | - | GCA_018140865.1 | ^109^ |
| KYI32 | South Korea | nuruk | - | GCA_043290115.1 | ^81^ |
| LBM30007 | Japan | soy sauce | - | GCA_022023695.1 | ^104^ |
| LBM30008 | Japan | soy sauce | H | GCA_022023705.1 | ^104^ |
| M2040 | South Korea | meju | C | SRR7615261 | ^110^ |
| MA1 | South Korea | doenjang meju | - | GCA_025738265.1 | ^111^ |
| MA2 | South Korea | doenjang meju | L | GCA_044231575.1 | ^81^ |
| NBRC 4078 | unknown | shoyu | Q | GCA_030270465.1 | ^103^ |
| NBRC 4079 | unknown | shoyu | Q | GCA_030270485.1 | ^103^ |
| NBRC 4191 | unknown | unknown | R | GCA_030270505.1 | ^103^ |
| NBRC 4228 | unknown | unknown | E | GCA_030270525.1 | ^103^ |
| NBRC 4377 | unknown | unknown | - | GCA_030270545.1 | ^103^ |
| NRRL 13765 | USA | amazake | G | TBD | ^112,113^ |
| NRRL 1911 | Japan | koji | F | GCA_034768135.1 | ^18,112^ |
| NRRL 1958 | South Pacific Ocean | optical mask | - | TBD | ^112,113^ |
| NRRL 1998 | unknown | koji | R | TBD | ^112,113^ |
| NRRL 2217 | China | soy sauce | - | TBD | ^112,113^ |
| NRRL 2218 | China | soy sauce | O | GCA_034768115.1 | ^18,112^ |
| NRRL 2220 | China | soy sauce | O | TBD | ^112,113^ |
| NRRL 31119 | Japan | amazake | S | TBD | ^112,113^ |

**Supplementary Table S1.** (continued)

| Strain ID | Country / region of isolation | Isolation source / use | Clade | Genome data source | Reference(s) |
| --- | --- | --- | --- | --- | --- |
| NRRL 32614 | Malaysia | soy sauce | O | GCA_034768095.1 | ^18,114^ |
| NRRL 32657 | Malaysia | soy sauce | N | TBD | ^113,114^ |
| NRRL 3483 | Japan | miso | E | GCA_034767915.1 | ^18,114^ |
| NRRL 3484 | Japan | miso | E | TBD | ^113,114^ |
| NRRL 3485 | Japan | miso | C | TBD | ^113,114^ |
| NRRL 3486 | Japan | miso | C | TBD | ^113,114^ |
| NRRL 3487 | Japan | miso | C | TBD | ^113,114^ |
| NRRL 3488 | Japan | miso | C | TBD | ^113,114^ |
| NRRL 3502 | unknown | unknown | C | TBD | ^112,113^ |
| NRRL 35191 | Colombia | coffee | - | TBD | ^115^ |
| NRRL 35739 | USA | soil | - | TBD | ^113^ |
| NRRL 35890 | Philippines | air/steamed rice | - | GCA_034767955.1 | ^18,112^ |
| NRRL 448 | unknown | unknown | F | TBD | ^112,113^ |
| NRRL 451 | China | soy sauce | F | TBD | ^112,113^ |
| NRRL 455 | Japan | koji | F | TBD | ^112,113^ |
| NRRL 456 | Japan | koji | F | TBD | ^112,113^ |
| NRRL 458 | Japan | koji | F | TBD | ^112,113^ |
| NRRL 459 | Japan | koji | - | TBD | ^112,113^ |
| NRRL 460 | Japan | koji | F | TBD | ^112,113^ |
| NRRL 466 | Japan | sake | S | TBD | ^112,113^ |
| NRRL 471 | Japan | sake | - | TBD | ^112,113^ |
| NRRL 476 | Japan | tamari | M | TBD | ^112,113^ |
| NRRL 480 | Japan | shoyu | - | TBD | ^112,113^ |
| NRRL 4803 | unknown | unknown | T | TBD | ^112,113^ |
| NRRL 481 | Japan | miso | T | TBD | ^113,114^ |
| NRRL 484 | USA | corn meal | - | TBD | ^112,113^ |
| NRRL 506 | USA | milk | - | TBD | ^112,113^ |
| NRRL 5588 | unknown | dust in soy sauce/miso factory | C | TBD | ^112,113^ |
| NRRL 5589 | Japan | defatted soybean meal | B | TBD | ^112,113^ |
| NRRL 5592 | Japan | sake | G | GCA_034767935.1 | ^18,112^ |
| NRRL 5593 | unknown | miso | C | TBD | ^112,113^ |
| NRRL 5938 | Japan | tane koji | F | TBD | ^112,113^ |
| NRRL 6270 | Taiwan | soy sauce | E | TBD | ^112,113^ |
| NRRL 6574 | Japan | hama natto | C | GCA_034767895.1 | ^18,112^ |
| NRRL 694 | unknown | silage | F | TBD | ^112,113^ |
| NRRL 695 | unknown | unknown | F | TBD | ^112,113^ |
| NRRL 696 | unknown | unknown | F | TBD | ^112,113^ |
| RIB1108 | Japan | miso | D | CAoGDX | ^82,116^ |
| RIB1133 | Japan | rice koji | - | CAoGDX | ^82,82^ |
| RIB1172 | Japan | koji | F | CAoGDX | ^82,117^ |
| RIB1178 | Japan | koji | - | CAoGDX | ^82,117^ |
| RIB1187 | Japan | koji | N | CAoGDX | ^82,117^ |
| RIB128 | Japan | sake | C | CAoGDX | ^82,82^ |

**Supplementary Table S1.** (continued)

| Strain ID | Country / region of isolation | Isolation source / use | Clade | Genome data source | Reference(s) |
| --- | --- | --- | --- | --- | --- |
| RIB143 | Japan | sake | A | CAoGDX | ^82,117^ |
| RIB163 | Japan | sake | G | CAoGDX | ^82,117^ |
| RIB301 | Japan | soy sauce | - | CAoGDX | ^82,117^ |
| RIB306 | Japan | soy sauce | M | CAoGDX | ^82,117^ |
| RIB312 | Japan | shoyu | P | CAoGDX | ^82,82^ |
| RIB315 | Japan | miso/soy sauce | C | CAoGDX | ^82,117^ |
| RIB326 | Japan | shoyu | A | GCA_000320905.1 | ^82,82^ |
| RIB331 | Japan | miso | C | SRR495609 | ^82,118^ |
| RIB333 | Japan | miso | C | CAoGDX | ^82,118^ |
| RIB338 | Japan | miso | C | CAoGDX | ^82,82^ |
| RIB40 | Japan | broad bean in the gutter of a soy sauce factory | F | GCF_000184455.2 | ^82,82^ |
| RIB430 | Japan | koji | S | CAoGDX | ^82,117^ |
| RIB632 | Japan | sake | G | SRR495763 | ^82,118^ |
| RIB642 | Japan | sake | C | SRR495765 | ^82,118^ |
| RIB802 | Japan | miso | C | CAoGDX | ^82,117^ |
| RIB915 | Japan | soy sauce | - | CAoGDX | ^82,117^ |
| RIB941 | Japan | shoyu | H | CAoGDX | ^82,82^ |
| RIB949 | Japan | soy sauce | - | SRR495766 | ^82,118^ |
| RIB954 | Japan | soy sauce | E | CAoGDX | ^82,82^ |
| RIBOIS01 | Japan | koji | A | CAoGDX | ^82,117^ |
| SRCM101975 | South Korea | meju | - | GCA_002214955.1 | ^119^ |
| SRCM101989 | South Korea | meju | - | GCA_002214965.1 | ^120^ |
| TK-1 | Japan | sake | A | GCA_009684795.1 | ^5^ |
| TK-10 | Japan | miso | G | GCA_009684975.1 | ^5^ |
| TK-11 | Japan | miso | F | GCA_009684995.1 | ^5^ |
| TK-12 | Japan | shoyu | H | GCA_009685015.1 | ^5^ |
| TK-13 | Japan | shoyu | E | GCA_009685035.1 | ^5^ |
| TK-14 | Japan | shoyu | H | GCA_009685055.1 | ^5^ |
| TK-15 | Japan | miso | G | GCA_009685075.1 | ^5^ |
| TK-16 | Japan | miso | C | GCA_009685095.1 | ^5^ |
| TK-17 | Japan | miso | A | GCA_009685115.1 | ^5^ |
| TK-18 | Japan | sake | A | GCA_009686465.1 | ^5^ |
| TK-19 | Japan | sake | F | GCA_009686485.1 | ^5^ |
| TK-2 | Japan | sake | A | GCA_009684815.1 | ^5^ |
| TK-20 | Japan | sake | B | GCA_009686505.1 | ^5^ |
| TK-21 | Japan | sake | F | GCA_009686525.1 | ^5^ |
| TK-22 | Japan | sake | A | GCA_009686545.1 | ^5^ |
| TK-23 | Japan | sake | C | GCA_009686565.1 | ^5^ |
| TK-24 | Japan | miso | R | GCA_009686585.1 | ^5^ |
| TK-25 | Japan | sake | C | GCA_009686605.1 | ^5^ |
| TK-26 | Japan | sake | B | GCA_009686625.1 | ^5^ |
| TK-27 | Japan | miso | G | GCA_009686645.1 | ^5^ |
| TK-28 | Japan | shoyu | E | GCA_009686665.1 | ^5^ |

**Supplementary Table S1.** (continued)

| Strain ID | Country / region of isolation | Isolation source / use | Clade | Genome data source | Reference(s) |
| --- | --- | --- | --- | --- | --- |
| TK-29 | Japan | shoyu | P | GCA_009686685.1 | ^5^ |
| TK-3 | Japan | sake | F | GCA_009684835.1 | ^5^ |
| TK-30 | Japan | sake | B | GCA_009686705.1 | ^5^ |
| TK-31 | Japan | sake | B | GCA_009686725.1 | ^5^ |
| TK-32 | Japan | sake | F | GCA_009686745.1 | ^5^ |
| TK-33 | Japan | sake | A | GCA_009686765.1 | ^5^ |
| TK-34 | Japan | sake | A | GCA_009686785.1 | ^5^ |
| TK-35 | Japan | sake | C | GCA_009686805.1 | ^5^ |
| TK-36 | Japan | sake | A | GCA_009686825.1 | ^5^ |
| TK-37 | Japan | sake | A | GCA_009686845.1 | ^5^ |
| TK-38 | Japan | sake | F | GCA_009686865.1 | ^5^ |
| TK-39 | Japan | other | B | GCA_009686885.1 | ^5^ |
| TK-4 | Japan | sake | F | GCA_009684855.1 | ^5^ |
| TK-40 | Japan | other | F | GCA_009686905.1 | ^5^ |
| TK-41 | Japan | miso | G | GCA_009686925.1 | ^5^ |
| TK-42 | Japan | miso | G | GCA_009686945.1 | ^5^ |
| TK-43 | Japan | miso | A | GCA_009686965.1 | ^5^ |
| TK-44 | Japan | miso | A | GCA_009686985.1 | ^5^ |
| TK-45 | Japan | miso | C | GCA_009687005.1 | ^5^ |
| TK-46 | Japan | miso | C | GCA_009687025.1 | ^5^ |
| TK-47 | Japan | miso | G | GCA_009687045.1 | ^5^ |
| TK-48 | Japan | shoyu | E | GCA_009687065.1 | ^5^ |
| TK-49 | Japan | shoyu | E | GCA_009687085.1 | ^5^ |
| TK-5 | Japan | sake | C | GCA_009684875.1 | ^5^ |
| TK-50 | Japan | shoyu | E | GCA_009687105.1 | ^5^ |
| TK-51 | Japan | sake/miso | A | GCA_009687125.1 | ^5^ |
| TK-52 | Japan | sake/miso | A | GCA_009687145.1 | ^5^ |
| TK-53 | Japan | sake/miso | A | GCA_009687165.1 | ^5^ |
| TK-54 | Japan | miso | C | GCA_009687185.1 | ^5^ |
| TK-55 | Japan | sake/miso | C | GCA_009687205.1 | ^5^ |
| TK-56 | Japan | miso | C | GCA_009687225.1 | ^5^ |
| TK-57 | Japan | sake | G | GCA_009687245.1 | ^5^ |
| TK-58 | Japan | sake | A | GCA_009687265.1 | ^5^ |
| TK-59 | Japan | shoyu | D | GCA_009687285.1 | ^5^ |
| TK-6 | Japan | sake | A | GCA_009684895.1 | ^5^ |
| TK-60 | Japan | shoyu | D | GCA_009687305.1 | ^5^ |
| TK-61 | Japan | sake/miso | A | GCA_009687325.1 | ^5^ |
| TK-62 | Japan | sake/miso | F | GCA_009687345.1 | ^5^ |
| TK-63 | Japan | sake | A | GCA_009687365.1 | ^5^ |
| TK-64 | Japan | sake | C | GCA_009687385.1 | ^5^ |
| TK-65 | Japan | sake | C | GCA_009687405.1 | ^5^ |
| TK-66 | Japan | shoyu | - | GCA_009687425.1 | ^5^ |
| TK-67 | Japan | shoyu | E | GCA_009687445.1 | ^5^ |

**Supplementary Table S1.** (continued)

| Strain ID | Country / region of isolation | Isolation source / use | Clade | Genome data source | Reference(s) |
| --- | --- | --- | --- | --- | --- |
| TK-68 | Japan | shoyu | E | GCA_009687465.1 | ^5^ |
| TK-69 | Japan | shoyu | E | GCA_009687485.1 | ^5^ |
| TK-7 | Japan | miso | C | GCA_009684915.1 | ^5^ |
| TK-70 | Japan | miso | C | GCA_009687505.1 | ^5^ |
| TK-71 | Japan | miso | G | GCA_009687525.1 | ^5^ |
| TK-72 | Japan | miso | A | GCA_009687545.1 | ^5^ |
| TK-73 | Japan | miso | A | GCA_009687565.1 | ^5^ |
| TK-74 | Japan | miso | C | GCA_009687585.1 | ^5^ |
| TK-75 | Japan | miso | C | GCA_009687605.1 | ^5^ |
| TK-76 | Japan | miso | C | GCA_009687625.1 | ^5^ |
| TK-77 | Japan | miso | A | GCA_009687645.1 | ^5^ |
| TK-78 | Japan | sake | A | GCA_009687665.1 | ^5^ |
| TK-79 | Japan | sake | A | GCA_009687685.1 | ^5^ |
| TK-8 | Japan | other | A | GCA_009684935.1 | ^5^ |
| TK-80 | Japan | sake | A | GCA_009687705.1 | ^5^ |
| TK-81 | Japan | sake | A | GCA_009687725.1 | ^5^ |
| TK-82 | Japan | sake | F | GCA_009687745.1 | ^5^ |
| TK-9 | Japan | shoyu | A | GCA_009684955.1 | ^5^ |

**Supplementary Table S2. Gap-filling reactions manually added to the *Aspergillus oryzae* pan-genome scale metabolic model (*pAo*).** These reactions were added to the *pAo* model to ensure flux balance analysis (FBA) can accurately simulate metabolic activity on specific substrates.

| ID | Name | Equation | Gene association | Lower bound | Upper bound |
| --- | --- | --- | --- | --- | --- |
| Gap_Arbutin_r1 | Arbutin hydrolysis | H2O[e] + Arbutin[e] => beta-D-glucose[e] + Hydroquinone[e] | cluster_3320 or cluster_2612 | 0 | 1000 |
| Gap_Arbutin_r2 | Hydroquinone transport | Hydroquinone[e] => Hydroquinone[c] |  | 0 | 1000 |
| Gap_Arbutin_r3 | Arbutin r3 | CO2[c] + Hydroquinone[c] => 2,5-Dihydroxybenzoate[c] |  | 0 | 1000 |
| Gap_Arbutin_r4 | Arbutin r4 | oxygen[c] + 2,5-Dihydroxybenzoate[c] => 3-Maleylpyruvate[c] |  | 0 | 1000 |
| Gap_Arbutin_r5 | Arbutin r5 | 3-Maleylpyruvate[c] => 3-Fumarylpyruvate[c] |  | 0 | 1000 |
| Gap_Arbutin_r6 | Arbutin r6 | 3-Fumarylpyruvate[c] => fumarate[c] + pyruvate[c] |  | 0 | 1000 |
| Gap_Rhamnose_r1 | Rhamnose uptake | L-Rhamnose[e] => L-Rhamnose[c] |  | 0 | 1000 |
| Gap_Rhamnose_r2 | Rhamnose r2 | NADP(+)[c] + L-Rhamnose[c] => NADPH[c] + L-rhamnono-1,4-lactone[c] |  | 0 | 1000 |
| Gap_Rhamnose_r4 | Rhamnose r4 | L-Rhamnonic acid[c] => H2O[c] + 2-Dehydro-3-deoxy-L-rhamnonate[c] |  | 0 | 1000 |
| Gap_Rhamnose_r5 | Rhamnose r5 | 2-Dehydro-3-deoxy-L-rhamnonate[c] => pyruvate[c] + (S)-lactaldehyde[c] |  | 0 | 1000 |
| Gap_4HPA_r1 | 4HPA uptake | 4-Hydroxyphenylacetate[e] => 4-Hydroxyphenylacetate[c] |  | 0 | 1000 |
| Gap_3HPA_r1 | 3HPA uptake | 3-Hydroxyphenylacetate[e] => 3-Hydroxyphenylacetate[c] |  | 0 | 1000 |
| Gap_3HPA_r2 | 3HPA r2 | NADPH[c] + oxygen[c] + 3-Hydroxyphenylacetate[c] => H2O[c] + homogentisate[c] + NADP(+)[c] |  | 0 | 1000 |
| Gap_L_Norvaline_r1 | Norvaline uptake | L-Norvaline[e] => L-Norvaline[c] |  | 0 | 1000 |
| Gap_L_Norvaline_r2 | Norvaline gap2 | L-Norvaline[c] => L-Norvaline[m] |  | 0 | 1000 |
| Gap_L_Norvaline_r3 | Norvaline gap3 | L-Norvaline[m] => NH3[m] + 2-oxopentanoate[m] |  | 0 | 1000 |
| Gap_L_Norvaline_r4 | Norvaline gap4 | coenzyme A[m] + 2-oxopentanoate[m] => CO2[m] + isobutyryl-CoA[m] |  | 0 | 1000 |
| Gap_L_Norvaline_r5 | Norvaline gap5 | H2O[m] + 2-Methylprop-2-enoyl-CoA[m] => (S)-3-Hydroxyisobutyryl-CoA[m] |  | 0 | 1000 |
| Gap_L_Norvaline_r6 | Norvaline gap6 | H2O[m] + (S)-3-Hydroxyisobutyryl-CoA[m] => coenzyme A[m] + (S)-3-Hydroxyisobutyrate[m] |  | 0 | 1000 |
| Gap_L_Norvaline_r7 | Norvaline gap7 | NAD(+)[m] + (S)-3-Hydroxyisobutyrate[m] => NADH[m] + (S)-Methylmalonate semialdehyde[m] |  | 0 | 1000 |
| Gap_L_Norvaline_r8 | Norvaline gap8 | H2O[m] + oxygen[m] + (S)-Methylmalonate semialdehyde[m] => hydrogen peroxide[m] + Methylmalonate[m] |  | 0 | 1000 |
| Gap_L_Norvaline_r9 | Norvaline gap9 | coenzyme A[m] + NAD(+)[m] + (S)-Methylmalonate semialdehyde[m] => NADH[m] + (R)-Methylmalonyl-CoA[m] |  | 0 | 1000 |
| Gap_L_Norvaline_r10 | Norvaline gap10 | (R)-Methylmalonyl-CoA[m] => succinyl-CoA[m] |  | 0 | 1000 |

**Supplementary Table S2.** (continued)

| ID | Name | Equation | Gene association | Lower bound | Upper bound |
| --- | --- | --- | --- | --- | --- |
| Gap_b_Gentiobiose_r1 | Gentiobiose gap1 | Gentiobiose[e] => 2 beta-D-glucose[e] | cluster_2603 | 0 | 1000 |
| Gap_Turanose_r1 | Turanose gap1 | Turanose[e] => D-fructose[e] + alpha-D-glucose[e] |  | 0 | 1000 |
| Gap_Palatinose_r1 | Palatinose gap1 | Palatinose[e] => D-fructose[e] + alpha-D-glucose[e] |  | 0 | 1000 |
| Gap_b_Cyclodextrin_r1 | b_Cyclodextrin gap1 | b_Cyclodextrin[e] => alpha-D-glucose[e] |  | 0 | 1000 |
| Gap_myo_Inositol_r1 | myo_Inositol gap1 | NAD(+)[c] + L-Gulonate[c] => NADH[c] + 2-keto-L-gulonate[c] |  | 0 | 1000 |
| Gap_myo_Inositol_r2 | myo_Inositol gap2 | NADH[c] + 2-keto-L-gulonate[c] => NAD(+)[c] + L-idonate[c] |  | 0 | 1000 |
| Gap_myo_Inositol_r3 | myo_Inositol gap3 | NADPH[c] + 2-keto-L-gulonate[c] => NADP(+)[c] + L-idonate[c] |  | 0 | 1000 |
| Gap_myo_Inositol_r4 | myo_Inositol gap4 | NAD(+)[c] + L-idonate[c] => NADH[c] + 5-keto-D-gluconate[c] |  | 0 | 1000 |
| Gap_myo_Inositol_r5 | myo_Inositol gap5 | NADH[c] + 5-keto-D-gluconate[c] => D-gluconate[c] + NAD(+)[c] |  | 0 | 1000 |
| Gap_Adonitol_r1 | Adonitol uptake | Adonitol[e] => Adonitol[c] |  | 0 | 1000 |
| Gap_Adonitol_r2 | Adonitol gap2 | NADP(+)[c] + Adonitol[c] => NADPH[c] + D-ribose[c] | cluster_6864 | 0 | 1000 |
| Gap_g_Cyclodextrin_r1 | g_Cyclodextrin gap1 | g_Cyclodextrin[e] => alpha-D-glucose[e] |  | 0 | 1000 |
| Gap_L_Ornithine_r1 | L_Ornithine uptake | L-ornithine[e] => L-ornithine[c] |  | 0 | 1000 |
| Gap_Putrescine_r1 | Putrescine uptake | putrescine[e] => putrescine[c] |  | 0 | 1000 |
| Gap_b_Phenylethylamine_r1 | b_Phenylethylamine uptake | Phenethylamine[e] => Phenethylamine[c] |  | 0 | 1000 |
| Gap_b_Phenylethylamine_r2 | b_Phenylethylamine gap2 | phenylacetate[c] <=> 4-Hydroxyphenylacetate[c] |  | -1000 | 1000 |
| Gap_Dihydroxy_Acetone_r1 | Dihydroxy_Acetone uptake | glycerone[e] => glycerone[c] |  | 0 | 1000 |
| Gap_Agmatine_r1 | Agmatine uptake | agmatine[e] => agmatine[c] |  | 0 | 1000 |
| Gap_L_Tryptophan_r1 | L_Tryptophan gap1 | anthranilate[c] + NADPH[c] + oxygen[c] => NH3[c] + NADP(+)[c] + 2,3-Dihydroxybenzoate[c] | cluster_7880 | 0 | 1000 |
| Gap_L_Tryptophan_r2 | L_Tryptophan gap2 | 2,3-Dihydroxybenzoate[c] => CO2[c] + Catechol[c] | cluster_12209 | 0 | 1000 |
| Gap_L_Tryptophan_r3 | L_Tryptophan gap3 | Catechol[c] => oxygen[c] + cis,cis-Muconate[c] | cluster_13016 | 0 | 1000 |
| Gap_L_Tryptophan_r4 | L_Tryptophan gap4 | cis,cis-Muconate[c] => Muconolactone[c] | cluster_11391 | 0 | 1000 |
| Gap_L_Tryptophan_r5 | L_Tryptophan gap5 | Muconolactone[c] => 3-Oxoadipate enol-lactone[c] | cluster_17315 | 0 | 1000 |
| Gap_L_Tryptophan_r6 | L_Tryptophan gap6 | H2O[c] + 3-Oxoadipate enol-lactone[c] => 3-Oxoadipate[c] | cluster_7045 | 0 | 1000 |
| Gap_L_Tryptophan_r7 | L_Tryptophan gap7 | 3-Oxoadipate[c] => 3-Oxoadipate[m] |  | 0 | 1000 |
| Gap_L_Tryptophan_r8 | L_Tryptophan gap8 | succinyl-CoA[m] + 3-Oxoadipate[m] => succinate[m] + 3-Oxoadipyl-CoA[m] | cluster_1445 or cluster_356 | 0 | 1000 |
| Gap_L_Tryptophan_r9 | L_Tryptophan gap9 | coenzyme A[m] + 3-Oxoadipyl-CoA[m] => acetyl-CoA[m] + succinyl-CoA[m] | cluster_1258 or cluster_5098 or cluster_9705 | 0 | 1000 |

**Supplementary Table S2.** (continued)

| ID | Name | Equation | Gene association | Lower bound | Upper bound |
| --- | --- | --- | --- | --- | --- |
| Gap_4HBA_r1 | 4HBA uptake | 4-hydroxybenzoate[e] => 4-hydroxybenzoate[c] |  | 0 | 1000 |
| Gap_4HBA_r2 | 4HBA gap2 | 4-hydroxybenzoate[c] => CO2[c] + Catechol[c] |  | 0 | 1000 |
| Gap_Xanthine_r1 | Xanthine gap1 | H2O[c] + oxygen[c] + Urate[c] => hydrogen peroxide[c] + 5-hydroxyisourate[c] |  | 0 | 1000 |
| Gap_Xanthine_r2 | Xanthine gap2 | glyoxylate[p] => glyoxylate[c] |  | 0 | 1000 |
| Gap_Xanthine_r3 | Xanthine gap3 | acetyl-CoA[c] + glyoxylate[c] + H2O[c] => coenzyme A[c] + (S)-malate[c] |  | 0 | 1000 |
| Gap_Sorbac_r1 | Sorbic_Acid uptake1 | Sorbic_Acid[e] => Sorbic_Acid[c] |  | 0 | 1000 |
| Gap_Sorbac_r2 | Sorbic_Acid uptake2 | Sorbic_Acid[c] => Sorbic_Acid[m] |  | 0 | 1000 |
| Gap_Sorbac_r3 | Sorbic_Acid gap3 | ATP[m] + coenzyme A[m] + Sorbic_Acid[m] => AMP[m] + phosphate[m] + Sorboyl_CoA[m] |  | 0 | 1000 |
| Gap_Sorbac_r4 | Sorbic_Acid gap4 | Sorboyl_CoA[m] => crotonoyl-CoA[m] + CO2[m] |  | 0 | 1000 |

**Supplementary Table S3. Growth parameters for *Aspergillus oryzae* strains on four carbon sources.** Data was obtained from 24-well plate cultivations, and represents mean of individual spline fits ± standard deviation for three biological replicates.

| Strain ID | Carbon source | Maximum biomass (AU) | Maximum growth rate (AU * h^-1^) | Time to maximum biomass (h) | Time to maximum growth rate (h) | N |
| --- | --- | --- | --- | --- | --- | --- |
| NRRL 2217 | Glucose | 22.6 ± 1.1 | 0.612 ± 0.034 | 71.5 ± 0 | 26.5 ± 2.9 | 3 |
| NRRL 2217 | Glycerol | 17.3 ± 1.4 | 0.652 ± 0.072 | 71.5 ± 0 | 25.3 ± 0.9 | 3 |
| NRRL 2217 | Maltose | 17.4 ± 0.6 | 0.501 ± 0.007 | 71.5 ± 0 | 25.5 ± 6 | 3 |
| NRRL 2217 | Xylose | 19.7 ± 1.1 | 0.51 ± 0.071 | 71.5 ± 0 | 32.9 ± 3.1 | 3 |
| NRRL 3483 | Glucose | 17.2 ± 6.7 | 0.711 ± 0.157 | 57.5 ± 12.2 | 35.8 ± 2 | 3 |
| NRRL 3483 | Glycerol | 9.9 ± 5.9 | 0.602 ± 0.166 | 58.7 ± 11.1 | 49.1 ± 11.8 | 3 |
| NRRL 3483 | Maltose | 17.2 ± 1.6 | 0.599 ± 0.02 | 71.5 ± 0 | 36.2 ± 2.3 | 3 |
| NRRL 3483 | Xylose | 17.8 ± 2.4 | 0.673 ± 0.167 | 59.2 ± 3.1 | 38 ± 2 | 3 |
| NRRL 3488 | Glucose | 19.4 ± 1.5 | 0.557 ± 0.052 | 68.3 ± 3.4 | 34.3 ± 1.1 | 3 |
| NRRL 3488 | Glycerol | 18.7 ± 1.8 | 0.757 ± 0.089 | 71 ± 0.8 | 31.4 ± 1 | 3 |
| NRRL 3488 | Maltose | 19.3 ± 4.2 | 0.576 ± 0.191 | 71 ± 0.8 | 36.1 ± 8 | 3 |
| NRRL 3488 | Xylose | 21.1 ± 2.8 | 0.554 ± 0.044 | 71.5 ± 0 | 59.5 ± 15 | 3 |
| NRRL 35890 | Glucose | 29.2 ± 1.2 | 0.999 ± 0.08 | 71.5 ± 0 | 28.5 ± 1.5 | 3 |
| NRRL 35890 | Glycerol | 9.9 ± 1.1 | 0.685 ± 0.082 | 60.4 ± 19.3 | 28.6 ± 0.7 | 3 |
| NRRL 35890 | Maltose | 23.2 ± 1.3 | 0.798 ± 0.093 | 71.5 ± 0 | 29.7 ± 3.2 | 3 |
| NRRL 35890 | Xylose | 25.8 ± 1.9 | 1.373 ± 0.307 | 71.5 ± 0 | 35.2 ± 0.4 | 3 |
| NRRL 471 | Glucose | 33.2 ± 4.2 | 0.744 ± 0.105 | 71.5 ± 0 | 42.3 ± 7.8 | 3 |
| NRRL 471 | Glycerol | 16.5 ± 0.6 | 0.537 ± 0.03 | 71.5 ± 0 | 31.1 ± 2.6 | 3 |
| NRRL 471 | Maltose | 27.6 ± 4.2 | 0.729 ± 0.057 | 71.5 ± 0 | 28.9 ± 2.5 | 3 |
| NRRL 471 | Xylose | 28.2 ± 1.9 | 0.76 ± 0.095 | 71.5 ± 0 | 37 ± 1.9 | 3 |
| NRRL 5589 | Glucose | 8 ± 0.5 | 0.254 ± 0.018 | 71.5 ± 0 | 30.8 ± 35.3 | 3 |
| NRRL 5589 | Glycerol | 6.3 ± 2.1 | 0.311 ± 0.062 | 58.3 ± 22.8 | 15.1 ± 0.4 | 3 |
| NRRL 5589 | Maltose | 6.8 ± 0.9 | 0.214 ± 0.05 | 71.5 ± 0 | 30.7 ± 33.2 | 3 |
| NRRL 5589 | Xylose | 6.6 ± 0.6 | 0.213 ± 0.113 | 71 ± 0.8 | 46.2 ± 40.1 | 3 |
| NRRL 5592 | Glucose | 16 ± 2.3 | 0.402 ± 0.075 | 71.5 ± 0 | 33.8 ± 5.4 | 3 |
| NRRL 5592 | Glycerol | 20.6 ± 7.1 | 0.659 ± 0.244 | 71.5 ± 0 | 31.7 ± 4.3 | 3 |
| NRRL 5592 | Maltose | 14.6 ± 2.6 | 0.416 ± 0.09 | 70.4 ± 1.9 | 45.6 ± 1.6 | 3 |
| NRRL 5592 | Xylose | 14.4 ± 3.7 | 0.349 ± 0.062 | 71.5 ± 0 | 30.8 ± 30 | 3 |
| RIB40 | Glucose | 16.5 ± 0 | 0.488 ± 0.055 | 71.5 ± 0 | 35.2 ± 3.2 | 3 |
| RIB40 | Glycerol | 17.9 ± 2.4 | 0.799 ± 0.145 | 67.3 ± 6.1 | 35.7 ± 1.2 | 3 |
| RIB40 | Maltose | 22.5 ± 1.9 | 0.609 ± 0.044 | 71.5 ± 0 | 32.8 ± 0.5 | 3 |
| RIB40 | Xylose | 15.9 ± 0.6 | 0.49 ± 0.046 | 71.5 ± 0 | 46 ± 1.3 | 3 |

**Supplementary Table S4. Network characteristics comparing the pan-GEM (*pAo*), *oryzae2008* (*iWV1364*), and the cordycepin-focused model (*iNR1684*) for *Aspergillus oryzae*.** Genes are counted as the number of unique orthologous gene clusters as obtained from the USEARCH algorithm (both with and without GPR), while the value with the asterisks indicates the number of individual genes for *oryzae2008*. Metabolites present in multiple compartments are either counted separately or as one (unique). GPR: Gene-protein-reaction rule.

| **Network characteristics** | ***pAo*** | ***oryzae2008*** | ***iNR1684*** |
| --- | --- | --- | --- |
| Genes | 1397 | 1160 (1346*) | 1467 (1684*) |
| Metabolites | 1824 (1312 unique) | 1104 (808 unique) | 1623 (1155 unique) |
| Reactions | 2025 | 1412 | 1947 |
| Biochemical reactions | 1652 | 1069 | 1371 |
| Cytoplasm | 1244 | 744 | 990 |
| Mitochondria | 220 | 170 | 229 |
| Peroxisome | 80 | 19 | 65 |
| Extracellular | 83 | 30 | 28 |
| Ambiguous / Multiple | 25 | 106 | 59 |
| Transport reactions | 211 | 185 | 301 |
| With GPR | 66 | 61 | 136 |
| Without GPR | 145 | 124 | 165 |
| Exchange reactions | 162 | 158 | 275 |

**Supplementary Table S5. Biomass composition of *Aspergillus oryzae* as used in the pan-GEM (*pAo*) and the 187 strain-specific models.** The biomass composition was adopted directly from *oryzae2008* (iWV1364)^49^, and is based on experimentally measured macromolecular contents reported in literature^73–76^. The composition was determined for *A. oryzae* A1560 grown on glucose with ammonia as nitrogen source at a specific growth rate (µ) of 0.1 h^-1^ and a specific glucose uptake rate of 1.12 mmol glucose * (g dry weight [g_DW_] * h^-1^)^-1^. The resulting biomass has an average molecular weight of 239.68 g * mol^-1^, an elemental composition of CH_1.5_O_0.53_N_0.19_P_0.005_S_0.01_, and a molecular weight per C-mole of 25.1 g * C-mol^-1^. Average molecular weights refer to monomers in polymer (g * mol^-1^). Content is given both as grams per 100 g dry weight and as weight fraction normalized to 100% excluding ash. Stoichiometric coefficients are from the biomass formation reaction (R_r1897) in units of mmol of monomers per g_DW_; the ATP coefficient represents the growth-associated maintenance energy (GAM) and carries no associated mass fraction.

| Biomass component | Average molecular weight (g * mol^-1^) | Content (g * 100 g_DW_^-1^) | Content (% (g/g_DW_), excluding ash) | Stoichiometric coefficient (mmol * g_DW_^-1^) |
| --- | --- | --- | --- | --- |
| Protein | | | | |
| Protein | 134.58 | 40.0 | 47.1 | 3.50075 |
| Carbohydrates | | | | |
| Glucan | 162.1 | 20.8 | 24.6 | 1.51453 |
| Chitin | 203.2 | 7.0 | 8.3 | 0.40759 |
| D-Mannitol | 182.2 | 3.3 | 3.9 | 0.21333 |
| Glycogen | 666.6 | 0.1 | 0.1 | 0.00212 |
| Glycerol | 92.1 | 0.7 | 0.8 | 0.08952 |
| Nucleic acids | | | | |
| RNA | 341.9 | 5.3 | 6.2 | 0.18259 |
| DNA | 332.3 | 0.8 | 0.9 | 0.02836 |
| Lipids | | | | |
| Phosphatidylcholine | 834.8 | 2.38 | 2.80 | 0.03356 |
| Triacylglycerol | 954.96 | 2.12 | 2.49 | 0.02617 |
| Phosphatidylethanolamine | 782.5 | 0.97 | 1.14 | 0.01468 |
| Free fatty acids | 301.31 | 0.35 | 0.41 | 0.01365 |
| Phosphatidylserine | 827.3 | 0.40 | 0.47 | 0.00564 |
| Phosphatidylamine | 755.24 | 0.58 | 0.68 | 0.00903 |
| Energy | | | | |
| ATP (growth-associated maintenance) | - | - | - | 49.0000 |
| Ash | | | | |
| Ash | - | 15.1 | - | - |
| Total (excl. ash) | - | 84.9 | 100 | - |
